# Leucine supplementation does not attenuate the decline in daily muscle protein synthesis rates or preserve leg muscle mass during leg immobilization in young or older adults: a double-blind randomized trial

**DOI:** 10.1016/j.ajcnut.2026.101205

**Published:** 2026-01-22

**Authors:** Tyler A Churchward-Venne, Philippe JM Pinckaers, Joey SJ Smeets, Gabriel Nasri Marzuca-Nassr, Stefan HM Gorissen, Cas J Fuchs, Joan M Senden, Joy PB Goessens, Annemie P Gijsen, Will KWH Wodzig, Luc JC van Loon

**Affiliations:** 1 NUTRIM Institute of Nutrition and Translational Research in Metabolism, Department of Human Biology, Maastricht University Medical Center+, Maastricht, The Netherlands; 2 Department of Kinesiology and Physical Education, McGill University, Montreal, QC, Canada; 3 Research Institute of the McGill University Health Centre, Montreal, QC, Canada; 4 Division of Geriatric Medicine, McGill University, Montreal, QC, Canada; 5 Central Diagnostic Laboratory, Maastricht University Medical Center+, Maastricht, The Netherlands

**Keywords:** leucine supplementation, limb immobilization, muscle protein synthesis, disuse muscle atrophy, skeletal muscle, young adults, older adults

## Abstract

**Background:**

Muscle disuse leads to muscle atrophy that has been attributed to declines in basal and postprandial muscle protein synthesis (MPS) rates. Leucine regulates MPS and may attenuate disuse-induced declines in MPS rates and muscle mass.

**Objectives:**

The purpose of this study was to evaluate the capacity of leucine supplementation to attenuate disuse-induced declines in MPS rates and muscle mass in young and older adults.

**Methods:**

In a randomized, double-blind, parallel-group design, 24 young (23 ± 4 y) and 24 older (69 ± 4 y) recreationally active adults (equal sex distribution) underwent 3 d of unilateral knee immobilization (leg casting) and received a leucine [group of adult study participants who supplemented with 5 g of leucine 3 × daily with each main meal during 3 d of unilateral knee immobilization by means of a full leg cast (LEU)] or energy-matched carbohydrate [group of adult study participants who supplemented with 5 g of carbohydrate 3 × daily with each main meal during 3 d of unilateral knee immobilization by means of a full leg cast (PLA)] supplement. Preimmobilization and postimmobilization, quadriceps muscle cross-sectional area (CSA) was assessed in the immobilized (IM) and nonimmobilized (NO-IM) leg by computed tomography. MPS rates were assessed in both legs during immobilization via ^2^H_2_O coupled with saliva, blood, and muscle biopsy sampling.

**Results:**

In young and older adults, MPS rates were ∼15% and ∼23% lower in the IM compared with NO-IM leg (1.28 ± 0.29 compared with 1.50 ± 0.26 and 1.10 ± 0.16 compared with 1.46 ± 0.28%/d, respectively; leg: both *P* < 0.001), with no differences between LEU compared with PLA treatments (treatment: *P* = 0.932 and *P* = 0.742, respectively). CSA decreased by ∼1.2% and ∼1.1% in the IM leg in young and older adults (from 7162 ± 1148 to 7076 ± 1129 mm^2^ and from 5813 ± 1092 to 5750 ± 1096 mm^2^, respectively; leg × time interaction: both *P* < 0.001), with no differences between LEU compared with PLA (treatment: *P* = 0.374 and *P* = 0.998). IM leg MPS rates were lower in older compared with young adults [difference: –0.18 (95% confidence interval: –0.31, –0.04) %/d; *P* = 0.013]. No differences were observed in the absolute (mm^2^) or relative (%) decline in CSA between young and older adults (both *P* > 0.05).

**Conclusions:**

Leucine supplementation does not attenuate the decline in daily MPS rates or muscle mass during short-term limb immobilization in young or older adults.

**Clinical Trial Register No. (Netherlands Trial Register)**: NL-OMON45771.

## Introduction

Periods of injury, trauma, illness, and/or disease are associated with physical inactivity and skeletal muscle disuse. Muscle disuse results in rapid decreases in muscle mass (i.e., disuse muscle atrophy) that are generally more pronounced in the lower-limb muscles [1]. In terms of the temporal nature of muscle atrophy after the onset of disuse (e.g., enforced bed rest or immobilization), the rate of muscle loss appears to be more rapid during the early stages of disuse [2]. Importantly, a number of studies [[3], [4], [5]] have reported substantial muscle loss within as little as 2 to 4 d of muscle disuse, a finding particularly relevant given that ∼3 d is the mean work absence due to flu-related illness [6]. In otherwise healthy humans, the decline in muscle mass in response to muscle disuse appears to be mainly driven by a decline in both basal postabsorptive [[7], [8], [9]], and postprandial [[9], [10], [11]] muscle protein synthesis (MPS) rates. Therefore, proanabolic strategies targeting the stimulation of MPS rates may prove to mitigate disuse-induced muscle atrophy.

The essential branched-chain amino acid leucine is a key nutrient regulator of the mechanistic target of rapamycin complex 1 (mTORC1) pathway [12,13], an intracellular signaling cascade known to regulate MPS and enhance cellular growth (for review, see Saxton and Sabatini [14]). Leucine ingestion alone (i.e., in the absence of provision of additional amino acid substrate) can robustly elevate MPS rates [15]. Furthermore, lower doses of dietary protein [16,17] or essential amino acids [18,19] enriched with leucine have been shown to elicit increases in MPS rates that are comparable with the rates observed after ingestion of much larger doses of protein (20–40 g). In addition to regulating MPS rates, evidence derived from rodent models also suggests that leucine administration may reduce muscle protein breakdown rates [[20], [21], [22]]. Consequently, leucine supplementation may represent an effective therapeutic strategy to maintain or even increase MPS rates during short-term periods of skeletal muscle disuse, and as such, attenuate disuse-induced muscle atrophy.

Aging is associated with a decline in skeletal muscle mass that proceeds at a rate of ∼1% to 2% per year in those aged ≥50 y [23]. Episodic periods of muscle disuse represent a major health concern for older adults due to their increased risk of developing sarcopenia [24]. Periods of muscle disuse are common in older adults (due to illness, disease, or hospitalization) and have been suggested to punctuate age-related muscle loss [25]. Young and older adults may respond differently to a period of muscle disuse, as studies have reported either reduced [[26], [27], [28], [29]], and even greater [10,30,31], muscle loss in older compared with young adults. There is also evidence for age-related differences in the capacity of leucine-enriched nutrient intake to stimulate postprandial MPS rates [32], with older adults requiring greater amounts of ingested leucine to stimulate postprandial MPS rates and, as such, to overcome age-related anabolic resistance.

The aim of the present study was to evaluate the capacity of thrice daily free leucine supplementation (5 g, 3 × daily) with each main meal [group of adult study participants who supplemented with 5 g of leucine 3 × daily with each main meal during 3 d of unilateral knee immobilization by means of a full leg cast (LEU)] to attenuate the decline in daily MPS rates and preserve muscle mass during short-term (3 d) unilateral lower-limb immobilization in both young and older adults as compared with an energy-matched carbohydrate supplement [group of adult study participants who supplemented with 5 g of carbohydrate 3 × daily with each main meal during 3 d of unilateral knee immobilization by means of a full leg cast (PLA)]. We hypothesized that 3 d of muscle disuse via unilateral lower-limb immobilization would result in a decrease in daily MPS rates and loss of leg muscle mass in both young and older adults. Furthermore, we hypothesized that leucine supplementation would attenuate the decline in daily MPS rates and, as such, preserve leg muscle mass in both young and older adults.

## Methods

### Participants and ethical approval

Twenty-four young [12 males/12 females; age: 23 ± 4 y; BMI: 22.9 ± 2.4 kg/m^2^ (mean ± SD)] and 24 older (12 males/12 females; age: 69 ± 4 y; BMI: 24.8 ± 2.5 kg/m^2^) healthy adults volunteered to participate in this randomized, double-blind, parallel-group study. Participants were excluded based on the following criteria: previous participation in a stable isotope tracer study within the last 5 y, lower limb and/or back injuries, a history of thrombosis/cardiovascular disease, use of anticoagulants, musculoskeletal/orthopedic disorders, currently performing structured resistance exercise training, use of corticosteroids, current use of protein supplements, diabetes (type I or II), use of tobacco products, pregnant, undergoing hormone replacement therapy, or use of third generation oral contraceptives. Participants were recruited through advertisements placed on dedicated bulletin boards within Maastricht University and in local newspapers from 8 April, 2016 to 22 February, 2017. The experimental test days were conducted at Maastricht University Medical Centre + (Maastricht, the Netherlands) between 25 April, 2016 and 3 April, 2017. All participants were informed about the purpose of the study, the experimental procedures, and potential risks before providing informed written consent. The procedures followed were in accordance with the ethical standards of the Medical Ethics Committee of Maastricht University Medical Center+ on human experimentation and in accordance with the Helsinki Declaration of 1975 as revised in October 2013. The study was approved by the Medical Ethical Committee of the Maastricht University Medical Center+, the Netherlands (METC 153055), and was independently monitored by the Clinical Trial Center Maastricht. The study was prospectively registered at the Netherlands Trial Register: (https://onderzoekmetmensen.nl/en/trial/45771).

### Preliminary testing

Participants aged 18 to 35 (young) and 60 to 80 (older) y with a BMI >18.5 kg/m^2^ and <30.0 kg/m^2^ underwent an initial screening session to assess, body mass, height, resting heart rate, and blood pressure. Participants were also instructed on the correct use of crutches and how to avoid bearing weight on the casted leg [immobilized (IM)] in preparation for the 3-d unilateral lower-limb immobilization period. Participants were deemed healthy based on their responses to a medical questionnaire and screening results. The preliminary testing and onset of the experiment (day –1) were separated by ≥5 d.

### Diet and physical activity

All participants were instructed to refrain from strenuous physical activity and alcohol consumption for 3 d before the first experimental visit (described below) until the end of the study. In addition, all participants were instructed to fill out food intake questionnaires and wear an omnidirectional accelerometer (Actical, Philips Respironics Inc.) on the waist to record both food intake and daily step-count for 3 d immediately before the first experimental visit and during the 3-d immobilization period. Food intake questionnaires were returned by the participants on day 3 after the immobilization period, and were checked for completeness and accuracy by a study team member in the presence of the participant in case any follow-up questions or verification were required. Dietary intake from the questionnaires was analyzed in consultation with a dietician using *Mijn Eetmeter* (https://mijn.voedingscentrum.nl/nl/eetmeter/), publicly available free online software available from the Dutch Health Council. On the evening before each experimental visit, all participants were provided with frozen prepackaged meals purchased from a local grocery store. The meals contained 55% energy as carbohydrate, 30% energy as fat, and 15% energy as protein, were standardized for all participants, and were selected in consultation with a dietician. Participants were instructed to consume the entire meal at home no later than 20:00, after which they were instructed to remain fasted until the following morning.

### Study design and experimental protocol

The present investigation was a randomized, controlled, double-blind, parallel-group study involving a bilateral leg protocol, in which 1 leg was IM while the contralateral leg (NO-IM) served as a cotemporal, within-participant, ambulatory control. Within each treatment and age group, half of the participants were randomly assigned to have their self-reported dominant leg casted, whereas the other half were randomly assigned to have their nondominant leg casted. Young (*n* = 24; 12 male/12 female) and older (*n* = 24; 12 male/12 female) participants were randomly allocated to either a leucine (*n* = 12 per age category; 6 male/6 female) or carbohydrate control (*n* = 12 per age category; 6 male/6 female) treatment group. The randomization procedure to allocate treatment group was done via a random-number generator (www.randomization.com) in 4 blocks of 12 (1 block for each sex age group). An investigator not directly affiliated with the study was responsible for the randomization. Beverage specifications are outlined under *Nutritional* *intervention*. To limit diurnal and intrasubject variation, all measures were carried out according to a standardized time schedule at the same time of day.

**FIGURE 1:**
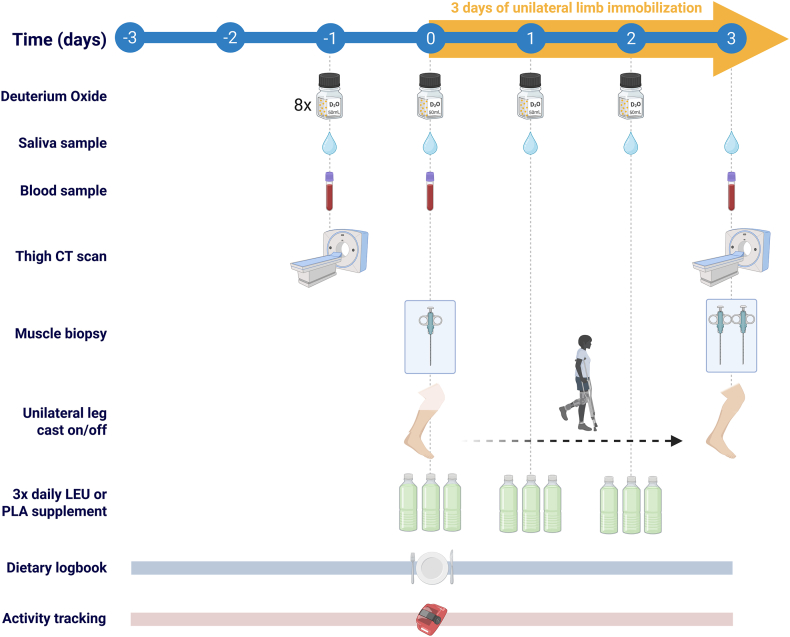
Schematic overview of the study design. CT, computed tomography; LEU, group of adult study participants who supplemented with 5 g of leucine 3 × daily with each main meal during 3 d of unilateral knee immobilization by means of a full leg cast; PLA, group of adult study participants who supplemented with 5 g of carbohydrate 3 × daily with each main meal during 3 d of unilateral knee immobilization by means of a full leg cast.

After preliminary testing, participants reported to the laboratory at ∼07:30 on 3 separate occasions in the overnight postabsorptive state (∼10 h). A schematic overview of the experimental design is depicted in Figure 1. During the first experimental visit (day –1), participants underwent deuterated water (D_2_O) loading after first providing a baseline venous blood and saliva sample. Participants ingested 8 × 50 mL (400 mL total) doses of 70% D_2_O (Cambridge Isotopes Laboratories) spaced 90 min apart [33] to minimize the risk of dizziness, and nausea in susceptible individuals [34]. During this visit, participants also underwent training/instruction on the correct use of crutches, and assessment of body composition [by dual-energy X-ray absorptiometry (DXA); Discovery A; Hologic] and bilateral upper-leg muscle cross-sectional area (CSA) [by computerized tomography (CT); described below].

The next morning (day 0), participants arrived at the laboratory at ∼07:30 in the overnight postabsorptive state and provided a baseline venous blood and saliva sample before undergoing a single skeletal muscle biopsy from the *m. vastus lateralis*. Participants were then outfitted with a full plaster leg cast to immobilize 1 leg (described below), provided with a set of crutches to remain ambulatory, and were discharged. During the 3-d immobilization period, participants collected a saliva sample using a cotton dental swab (to determine body water ^2^H enrichments) each evening and ingested 1 × 50 mL maintenance dose of 70% D_2_O every morning until cast removal. The participants were instructed to not eat or drink anything 30 min before saliva collection. During the immobilization period, participants also ingested their randomly assigned nutritional treatment (5 g leucine or carbohydrate) 3 × daily with each main meal (described below).

After the 3-d immobilization period (day 3), participants returned to the laboratory at ∼07:30 in the morning in the overnight postabsorptive state, had their cast removed, and underwent bilateral CT scans to assess changes in the CSA of the upper-leg muscles in both the IM and nonimmobilized (NO-IM) lower limbs. After the CT scans, the participants were transported to the laboratory via wheelchair where a baseline venous blood and saliva sample were collected before undergoing bilateral skeletal muscle biopsies from the *m. vastus lateralis* of both legs. All venous blood samples were collected into EDTA-containing tubes and centrifuged at 1000 × *g* for 10 min at 4°C. Aliquots of plasma were frozen in liquid nitrogen and stored at −80°C. Biopsy samples were collected using a 5-mm Bergström needle custom-adapted for manual suction [35]. Samples were obtained from separate incisions from the middle region of the *m. vastus lateralis*, ∼15 cm above the patella and ∼3 cm below entry through the fascia, under 1% xylocaine local anesthesia with adrenaline (1:100,000). Muscle samples were freed from any visible nonmuscle material, immediately frozen in liquid nitrogen, and stored at −80°C until further analysis. When the experimental protocol was complete, participants were provided with food and assessed for ∼30 min before leaving the laboratory.

### Skeletal muscle mass

The anatomical CSA of the quadriceps muscles was assessed via a single-slice CT scan (Philips Brilliance 64; Philips Medical Systems). During the scan, participants were placed in a supine position with their legs extended and their feet secured before obtaining a 3-mm thick axial image 15 cm above the top of the patella. The exact scanning location was marked with semipermanent ink on the participants legs to enable replication of the scan site after cast removal. ImageJ software (version 1.46r and 2.0.0; NIH) was used to analyze the CT scan images via manual tracing to determine the CSA of the quadriceps muscles in each leg.

### Limb immobilization protocol

Immediately after the baseline skeletal muscle biopsy (day 0), participants were taken via wheelchair to the Casting Room at the Maastricht University Medical Centre+, to have a full plaster leg cast applied. Application of the plaster leg cast marked the onset of the 3-d immobilization period. The leg cast was applied to the nonbiopsied limb, which was randomly assigned and counterbalanced for dominant and nondominant leg and extended from ∼5 cm above the ankle to ∼25 cm above the knee. The leg was cast at ∼30° of knee flexion to prevent participants from performing weight-bearing activities with the IM. Participants received crutches to remain ambulatory and were again instructed on their correct usage. The cast was removed at ∼08:00 after 3 d of immobilization, after which participants were transported by wheelchair until collection of the postimmobilization muscle biopsies.

### Nutritional intervention

During the immobilization period, participants received opaque plastic bottles containing either 5 g powdered leucine (Fusil, unflavored pharmaceutical grade instantized L-leucine powder, Ajinomoto North America) or 5 g powdered carbohydrate as maltodextrine (AVEBE) to ingest with each main meal (15 g daily: 5 g at breakfast, lunch, and dinner). The opaque bottles were visually identical, and also contained 2 calorie-free sweeteners (Natrena) to match the taste and smell of the nutritional treatments. Participants were instructed to add water to the bottle up to an indicated black line corresponding to 200 mL, shake, and ingest the supplement. Participants were required to note down the time of consumption and return all bottles to the study investigators to determine compliance with the dietary supplements.

### Body water ^2^H enrichment analyses

Enrichment of the body water pool was analyzed using saliva samples collected during the experimental protocol. Samples were thawed, centrifuged at 10,000 × *g*, and diluted 70-fold with ddH_2_O to achieve deuterium enrichments within the detection limits of the gas chromatograph-combustion-isotope ratio mass spectrometer (GC-C-IRMS). After dilution, samples were prepared for analysis on a GC-C-IRMS using the protocol of Scrimgeour and colleagues [36]. Briefly, small plastic cups holding 4 mg of catalyst (5% platinum on alumina, 325 mesh; Sigma-Aldrich) were placed inside 3-mL glass vials (Labco Exetainer; Labco). Three-hundred μL of diluted saliva sample were then transferred into the vials. The glass vials were sealed using rubber septums and a screw cap. Air in each vial was simultaneously evacuated and replaced by hydrogen gas. The prepared vials were left at 21°C for 24 h for deuterium equilibration to occur between the hydrogen gas and the saliva samples. The deuterium enrichment of the hydrogen gas was then measured in duplicate on a GC-C-IRMS (Micromass Optima IRMS fitted with a Multiprep and Gilson autoinjector; Micromass). Standard regression curves were applied from a series of known standard enrichment values against the measured values to assess the linearity of the mass spectrometer and to account for deuterium loss during equilibration.

### Plasma free [^2^H]-alanine enrichment analysis

Plasma amino acid enrichments were determined by GC-MS (Agilent 5975C MSD & 7890A GC). Plasma samples were deproteinized with dry 5-sulfosalicylic acid. Free amino acids were purified using cation exchange chromatography (AG 50W-X8 resin, mesh size: 100–200 μm, ionic form: hydrogen; Bio-Rad Laboratories). The purified amino acids were converted into tert-butyldimethylsilyl (tert-BDMS) derivatives with N-tert-butyldimethylsilyl-N-methyltrifluoroacetamide (MTBSTFA) before analysis by GC-MS. The plasma free alanine mass isotopomers (M and M+1) were determined using selective ion monitoring at *m*/*z* 232 and 233. Standard regression curves were applied from a series of known standard enrichment values against the measured values to assess the linearity of the mass spectrometer and to account for any isotope fractionation.

### Muscle protein-bound [^2^H]-alanine enrichment analysis

Mixed muscle protein-bound [^2^H]-alanine enrichments were analyzed in ∼60 mg wet muscle tissue. The sample was freeze dried, after which collagen, blood, and other nonmuscle fiber material were removed from the muscle fibers under a light microscope. The isolated muscle fiber mass was weighed, and 7 volumes of ice-cold 2% perchloric acid were added. The tissue was then sonicated and centrifuged. The supernatant was collected and processed in the same manner as the plasma samples, such that intracellular free [^2^H]-alanine enrichments could be measured by using their *t*-butyldimethylsilyl derivatives on a GC-MS. The protein pellet was washed with 3 additional 1.5-mL washes of 2% perchloric acid, dried, and hydrolyzed in 6 M HCl at 120°C for 15 to 18 h. The hydrolyzed protein fraction was dried under a nitrogen stream while being heated at 120°C. After being dissolved with a 50% acetic acid solution, samples were passed over Dowex exchange resin (AG 50W-X8, 100–200 mesh hydrogen form; Bio-Rad) by using 2 M NH_4_OH. Thereafter, the eluate was dried, and the purified amino acids were derivatized to their N(O,S)-ethoxycarbonyl ethyl esters [37]. The derivatized samples were measured using a gas chromatography-isotope ratio mass spectrometer (MAT 253; Thermo Fisher Scientific) equipped with a pyrolysis oven using a 60-m DB-17MS column and 5-m precolumn (number 122-4762; Agilent) and GC-Isolink. Ion masses 2 and 3 were monitored to determine the ^2^H/^1^H ratios of muscle protein-bound alanine. A series of known standards was applied to assess linearity of the mass spectrometer and to control for the loss of tracer.

### Calculations

Mixed muscle protein fractional synthesis rates (FSR) using the orally ingested D_2_O tracer were determined in both the IM and NO-IM leg based on the incorporation of [^2^H]-alanine into mixed muscle proteins and either the mean free [^2^H]-alanine enrichment in plasma or mean deuterium enrichment in saliva (as a surrogate for body water) as the precursor using the standard precursor-product equation expressed as daily rates as follows:

$$FSR=\left(\frac{\%}{day}\right)=\left(\frac{\;E_{M2}-E_{M1}}{E_{precursor}\times t\;}\right)\times 100\%$$

Where E_m1_ and E_m2_ are the mixed muscle protein-bound enrichments on days 0 and 3, respectively. E_precursor_ represents either the mean (over days 0 and 3) plasma free [^2^H]-alanine, or the mean (over days 0, 1, 2, and 3) body water deuterium enrichment corrected by a factor of 3.7 based on the mean number of deuterium moieties incorporated per alanine during de novo alanine synthesis [33,38], and *t* represents the time between biopsies (days 0 and 3).

### Outcome measures

The primary outcome measure in this study was daily mixed MPS rates [i.e., daily mixed muscle FSR (%/d)] over the 3-d immobilization period based on the application of D_2_O and measurement of protein-bound ^2^H-alanine enrichments in collected muscle samples. Quadriceps muscle size (based on CT-derived CSA in mm^2^) was a secondary outcome measure. All other outcome measures were tertiary.

### Statistical analysis

Participant’s characteristics were summarized via descriptive statistics (mean ± SD). Participant’s self-reported dietary intake and accelerometer-derived daily step-count before (i.e., at baseline) and during the 3-d immobilization period were analyzed separately in young and older adults via a 2-factor (time × treatment) repeated-measures analysis of variance (ANOVA) with time as a within-participant factor and treatment as a between-participant factor. Precursor pool isotope enrichments in blood plasma (free ^2^H-alanine) and body water (^2^H in saliva) were analyzed separately in young and older adults. In young adults, a 2-factor (time × treatment) repeated-measures ANOVA with time as a within-participant factor and treatment as a between-participant factor was applied. In older adults, a mixed effects model was employed due to a limited number of missing blood or saliva samples. Mixed muscle protein FSR (%/d) was first analyzed separately in young and older adults via a 2-factor (leg × treatment) repeated-measures ANOVA with leg as a within-participant factor and treatment as a between-participant factor. To evaluate the effect of age irrespective of treatment on mixed muscle protein FSR (%/d), data were collapsed across treatment groups for post hoc analysis and analyzed via a 2-factor (leg × age group) repeated-measures ANOVA with leg as a within-participant factor and age group as a between-participant factor. Quadriceps muscle size (CSA in mm^2^) was analyzed separately in young and older adults via a 3-factor (leg × time × treatment) repeated-measures ANOVA with leg and time as within-participant factors and treatment as a between-participant factor. To evaluate age-related differences, data were also collapsed across treatment groups for post hoc analysis, and CSA was analyzed via a 3-factor (leg × time × age group) repeated-measures ANOVA with leg and time as within-participant factors and age group as a between-participant factor. An independent samples *t*-test was performed to examine the difference in mixed muscle protein FSR between the IM leg and NO-IM leg between age groups. An independent samples *t*-test was also performed to examine the difference between both the absolute and relative change in quadriceps muscle CSA in the IM leg between age groups. Bonferroni post hoc tests were performed to control for type I error when performing multiple comparison testing. The Mauchley test was applied to evaluate the assumption of sphericity. If a significant Mauchley test was determined, the Greenhouse–Geisser correction factor was used to adjust the degrees of freedom accordingly. Pearson correlation coefficient analysis was applied post hoc to examine associations between baseline quadriceps muscle size (CSA in mm^2^) and the relative (%) and absolute (in mm^2^) change in quadriceps muscle CSA in the IM leg. In all statistical analyses, statistical significance was set at *P* < 0.05. Data are expressed as mean ± SD, mean difference with corresponding 95% confidence interval (CI) of the mean difference, along with individual participant data where appropriate. All analyses were performed using IBM SPSS statistics for Windows (version 27, IBM Corp) and GraphPad Prism (GraphPad Software, v.8.4.3).

A sample size calculation for a 2-tailed unpaired *t*-test was performed to detect a difference in means between LEU and PLA treatment groups in the IM leg for the primary outcome measure, FSR of MPS. On the basis of previous work, we used 1.2%/d as an estimate of steady-state diurnal MPS rates in the NO-IM leg. In response to muscle disuse, the total diurnal mean decline in MPS across postabsorptive and postprandial periods in the IM leg was estimated to be ∼50% (i.e., reduced to 0.6%/d) in the PLA group, but fully attenuated in the LEU group. Using G∗Power software, we determined that with a significance (*α*) of 0.05, an effect size (*d*) of 1.5, and a SD of 0.4%/d in both groups, a sample size of 11 participants per group would be sufficient to detect a between-group difference in FSR of 0.6%/d in the IM leg with a power (1 − *β*) > 0.90. To account for potential dropouts, we recruited 12 participants per group within each age category.

## Results

### Participants’ characteristics

Baseline characteristics of the young and older participants who were randomly assigned into LEU or PLA study arms and completed the trial are presented in Table 1. Of the 84 participants assessed for eligibility, 13 did not meet the inclusion criteria and were excluded from participation, and 18 discontinued participation. To meet the a priori sample size goal of *n* = 48 participants, 28 young and 25 older adults were randomly assigned. After 5 dropouts, the resulting sample available for complete analyses matched the planned *n* = 48. A CONSORT flow diagram is shown in Supplemental Figure 1.

**TABLE 1:** Characteristics of young and older adults supplemented with 5 g of carbohydrate (PLA) or 5 g of leucine (LEU) 3 × daily with each main meal during 3 d of unilateral knee immobilization

	Young		Older	
	2PLA	2LEU	2PLA	2LEU
1Age (y)	23 ± 5	23 ± 4	69 ± 4	69 ± 4
1Height (m)	1.75 ± 0.12	1.71 ± 0.09	1.68 ± 0.08	1.69 ± 0.09
1Weight (kg)	70.6 ± 12.4	67.2 ± 9.9	71.3 ± 11.6	70.5 ± 8.7
1BMI (kg/m^2^)	22.8 ± 1.9	23.1 ± 2.9	25.1 ± 2.7	24.5 ± 2.4
1Whole-body fat + bone-free mass (kg)	54.4 ± 11.1	49.7 ± 8.3	51.0 ± 10.1	49.9 ± 9.7
1Immobilized leg lean mass (kg)	9.9 ± 1.9	8.9 ± 1.5	8.6 ± 1.6	8.4 ± 1.8
1Whole-body fat (%)	20.4 ± 7.0	22.8 ± 6.1	25.9 ± 7.8	26.5 ± 9.2
1Systolic blood pressure (mmHg)	116 ± 13	117 ± 11	134 ± 10	140 ± 17
1Diastolic blood pressure (mmHg)	65 ± 8	66 ± 9	74 ± 9	78 ± 12
1Resting heart rate (bpm)	68 ± 6	75 ± 8	63 ± 12	65 ± 11

Abbreviations: LEU, group of adult study participants who supplemented with 5 g of leucine 3 × daily with each main meal during 3 d of unilateral knee immobilization by means of a full leg cast; PLA, group of adult study participants who supplemented with 5 g of carbohydrate 3 × daily with each main meal during 3 d of unilateral knee immobilization by means of a full leg cast.

1 Values are mean ± SD.

2 *n* = 12 per group.

### Dietary intake and physical activity

Self-reported nutritional intake and accelerometer-derived step-count in young and older adults during the 3-d baseline and 3-d immobilization period are shown in Supplemental Table 1. In young adults, there were no differences in mean energy (kJ) intake, carbohydrate, fat, or protein (g) intake, relative protein (g/kg/body mass) intake, or percent energy intake from carbohydrate, fat, or protein (%) between baseline and immobilization periods (time: all *P* > 0.05) or between LEU and PLA treatment groups (treatment: all *P* > 0.05). Immobilization resulted in a pronounced decline in habitual physical activity, with a decrease in step-count compared with the 3-d baseline period in both PLA [difference: –6053 (95% CI: –7986, –4119) steps; *P* < 0.001] and LEU, respectively [difference: –3112 (95% CI: –4877, –1347) steps; *P* = 0.001].

In older adults, there were no differences in mean energy (kJ) intake, carbohydrate, fat, or protein (g) intake, relative protein (g/kg/body mass) intake, or percent energy intake from carbohydrate, or fat (%) between baseline and immobilization periods (time: all *P* > 0.05) or between LEU and PLA treatment groups (treatment: all *P* > 0.05). Immobilization resulted in a pronounced decline in habitual physical activity, with a decrease in step-count compared with the 3-d baseline period in both PLA [difference: –6844 (95% CI: –8636, –5052); *P* < 0.001] and LEU [difference: –6928 (95% CI: –8720, –5136); *P* < 0.001].

### Precursor pool isotope enrichments

**FIGURE 2:**
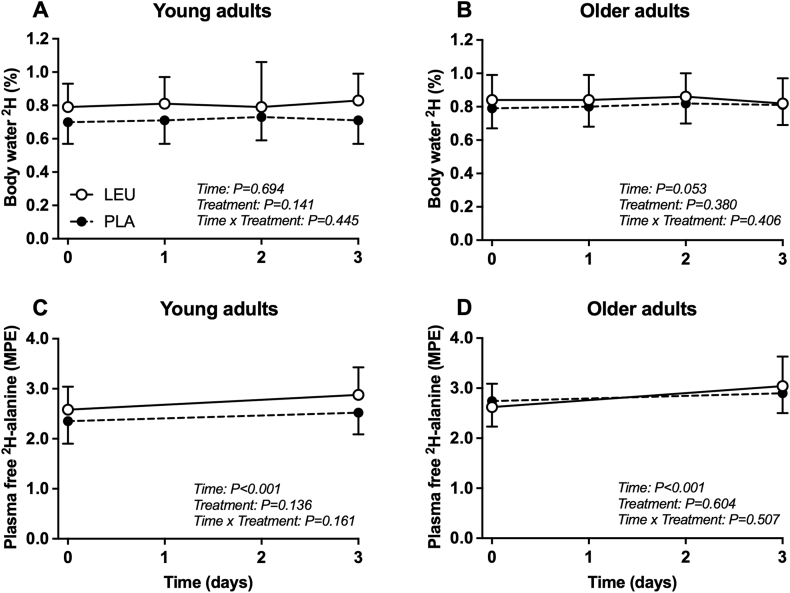
Body water ^2^H enrichments (%) (A–B) and plasma free ^2^H-alanine enrichments (MPE) (C–D) in young and older adult study participants who supplemented with 5 g of leucine (LEU) or 5 g of carbohydrate (PLA) 3 × daily with each main meal during 3 d of unilateral knee immobilization by means of a full leg cast. Values represent means ± SD, *n* = 12 per treatment group in each age group. Data were analyzed separately in young and older adult study participants with a 2-factor (treatment × time) repeated-measures ANOVA (young) and mixed effects model (older). ANOVA, analysis of variance; LEU, group of adult study participants who supplemented with 5 g of leucine 3 × daily with each main meal during 3 d of unilateral knee immobilization by means of a full leg cast; MPE, mole percent excess; PLA, group of adult study participants who supplemented with 5 g of carbohydrate 3 × daily with each main meal during 3 d of unilateral knee immobilization by means of a full leg cast.

Body water ^2^H enrichments (%) analyzed in collected saliva samples of young and older adult participants are shown in Figure 2A–B. In young adults, body water ^2^H enrichments on day 0 at the onset of the immobilization period were 0.70 ± 0.13% in PLA and 0.79 ± 0.14% in LEU. Body water ^2^H enrichments did not differ between days 0, 1, 2, and 3 (time: *P* = 0.694). In older adults, body water ^2^H enrichments on day 0 at the onset of the immobilization period were 0.79 ± 0.12% in PLA and 0.84 ± 0.15% in LEU. Body water ^2^H enrichments did not differ between days 0, 1, 2, and 3 (Time: *P* = 0.053).

Plasma free ^2^H-alanine enrichments [mole percent excess (MPE)] in young and older adults are shown in Figure 2C–D. In young adults, plasma free ^2^H-alanine enrichments (MPE) on day 0 at the onset of the immobilization period were 2.35 ± 0.45 in PLA and 2.58 ± 0.46 in LEU. On day 3 following the immobilization period, plasma free ^2^H-alanine enrichments (MPE) increased to 2.53 ± 0.43 in PLA and 2.88 ± 0.55 in LEU (Time: *P* < 0.001). In older adults, plasma free ^2^H-alanine enrichments (MPE) on day 0 at the onset of the immobilization period were 2.74 ± 0.50 in PLA and 2.62 ± 0.47 in LEU. On day 3 following the immobilization period, plasma free ^2^H-alanine enrichments (MPE) increased to 2.90 ± 0.40 in PLA and 3.04 ± 0.59 in LEU (Time: *P* < 0.001).

### Mixed muscle protein FSR

**FIGURE 3:**
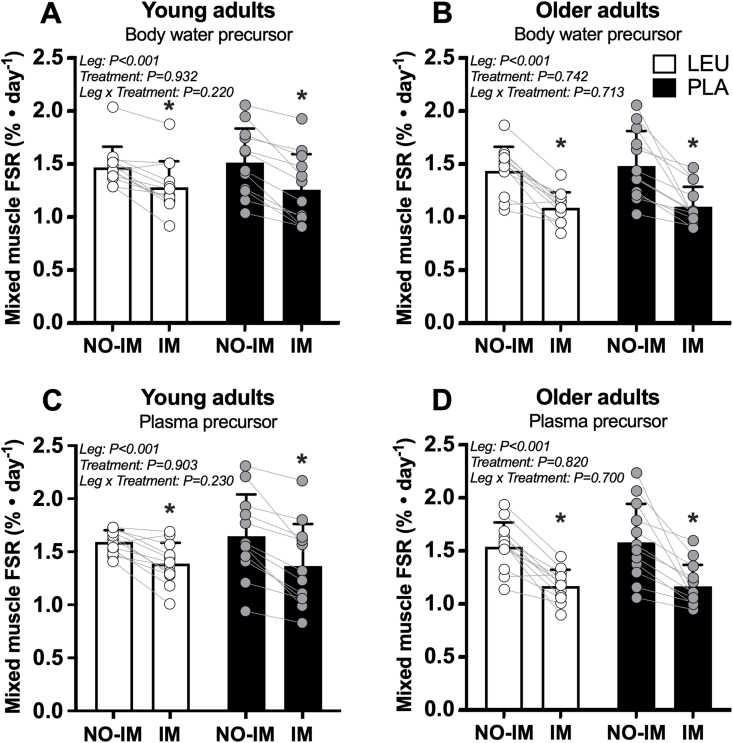
Mixed muscle FSR (%/d) calculated using body water ^2^H enrichments (A–B) and plasma free ^2^H-alanine enrichments (C–D) in young and older adult study participants who supplemented with 5 g of leucine (LEU) or 5 g of carbohydrate (PLA) 3 × daily with each main meal during 3 d of unilateral knee immobilization by means of a full leg cast. Values represent means ± SD and individual participant data, *n* = 12 per treatment group in each age group. Data were analyzed separately in young and older adult study participants with a 2-factor (leg × treatment) repeated-measures ANOVA. ∗Significant main effect for leg. ANOVA, analysis of variance; FSR, fractional synthesis rate; IM, immobilized leg; LEU, group of adult study participants who supplemented with 5 g of leucine 3 × daily with each main meal during 3 d of unilateral knee immobilization by means of a full leg cast; NO-IM, nonimmobilized leg; PLA, group of adult study participants who supplemented with 5 g of carbohydrate 3 × daily with each main meal during 3 d of unilateral knee immobilization by means of a full leg cast.

Mixed muscle protein FSR (%/d) calculated using body water ^2^H enrichments as the precursor pool compared between LEU and PLA treatments in young and older adult participants are shown in Figure 3A–B. In young adults, there was a main effect of leg, whereby mixed muscle protein FSR (%/d) in the IM leg (total: 1.28 ± 0.29%/d) was lower compared with the NO-IM (total: 1.50 ± 0.26%/d) leg [difference: –0.22 (95% CI: –0.28, –0.16) %/d; *P* < 0.001]. There was no effect of treatment (treatment: *P* = 0.932) or leg × treatment interaction (interaction: *P* = 0.220). In older adults, there was a main effect of leg, whereby mixed muscle protein FSR (%/d) in the IM leg (total: 1.10 ± 0.16%/d) was lower compared with the NO-IM (total: 1.46 ± 0.28%/d) leg [difference: –0.37 (95% CI: –0.47, –0.26) %/d; *P* < 0.001]. There was no effect of treatment (treatment: *P* = 0.742) or leg × treatment interaction (interaction: *P* = 0.713).

Mixed muscle protein FSR (%/d) calculated using plasma free ^2^H-alanine enrichments (MPE) as the precursor pool compared between LEU and PLA treatments in young and older adult participants is shown in Figure 3C–D. In young adults, there was a main effect of leg, whereby mixed muscle protein FSR (%/d) in the IM leg (total: 1.38 ± 0.30%/d) was lower compared with the NO-IM (total: 1.62 ± 0.28%/d) leg [difference: –0.24 (95% CI: –0.31, –0.17) %/d; *P* < 0.001]. There was no effect of treatment (treatment: *P* = 0.903) or leg × treatment interaction (interaction: *P* = 0.230). In older adults, there was a main effect of leg, whereby mixed muscle protein FSR (%/d) in the IM leg (total: 1.17 ± 0.17%/d) was lower compared with the NO-IM (total: 1.56 ± 0.30%/d) leg [difference: –0.39 (95% CI: –0.50, –0.28) %/d; *P* < 0.001]. There was no effect of treatment (treatment: *P* = 0.820) or leg × treatment interaction (interaction: *P* = 0.700).

**FIGURE 4:**
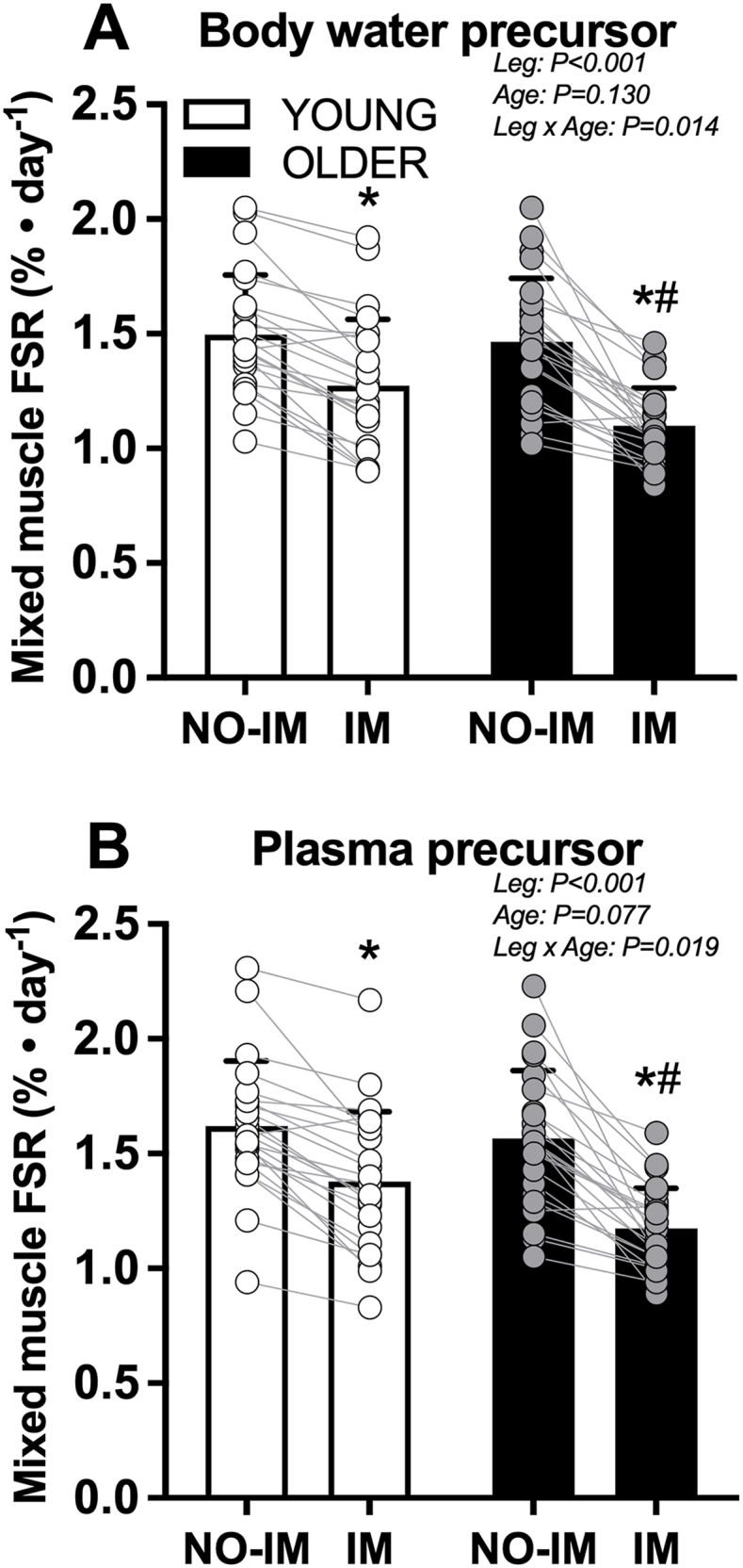
Mixed muscle FSR (%/d) calculated using body water ^2^H enrichments (A) and plasma free ^2^H-alanine enrichments (B) in young and older adult study participants during 3 d of unilateral knee immobilization by means of a full leg cast. In each age group, data were collapsed across treatment groups who supplemented with 5 g of leucine or 5 g of carbohydrate 3 × daily with each main meal. Values represent means ± SD and individual participant data, *n* = 24 per age group. Data were analyzed with a 2-factor (leg × age group) repeated-measures ANOVA. Bonferroni post hoc tests were performed to determine the difference between age groups within each leg and the difference between legs within each age group. ∗Significantly different from NO-IM leg within that age group. #Significantly different from Young IM leg. ANOVA, analysis of variance; FSR, fractional synthesis rate; IM, immobilized leg; NO-IM, nonimmobilized leg.

Mixed muscle protein FSR (%/d) calculated using body water ^2^H and plasma free ^2^H-alanine enrichments as the precursor pool compared between young and older adult participants are shown in Figure 4A–B (*n* = 24 per group, PLA and LEU combined). Mixed muscle protein FSR (%/d) calculated using body water ^2^H enrichments demonstrated a leg × age group interaction (*P* = 0.014). There were no differences in mixed muscle protein FSR between age groups in the NO-IM leg; however, mixed muscle protein FSR in the IM leg were lower in older compared with young adults [difference: –0.18 (95% CI: –0.31, –0.04) %/d; *P* = 0.013]. The difference in mixed muscle protein FSR between the IM leg and NO-IM leg was greater in older (–0.37 ± 0.24%/d) when compared with young (–0.22 ± 0.14%/d) adults [difference: 0.14 (95% CI: 0.03, 0.26) %/d; *P* = 0.014]. Similar results were obtained when using plasma free ^2^H-alanine enrichments as the precursor pool for calculating mixed muscle protein FSR.

### Quadriceps muscle CSA

**FIGURE 5:**
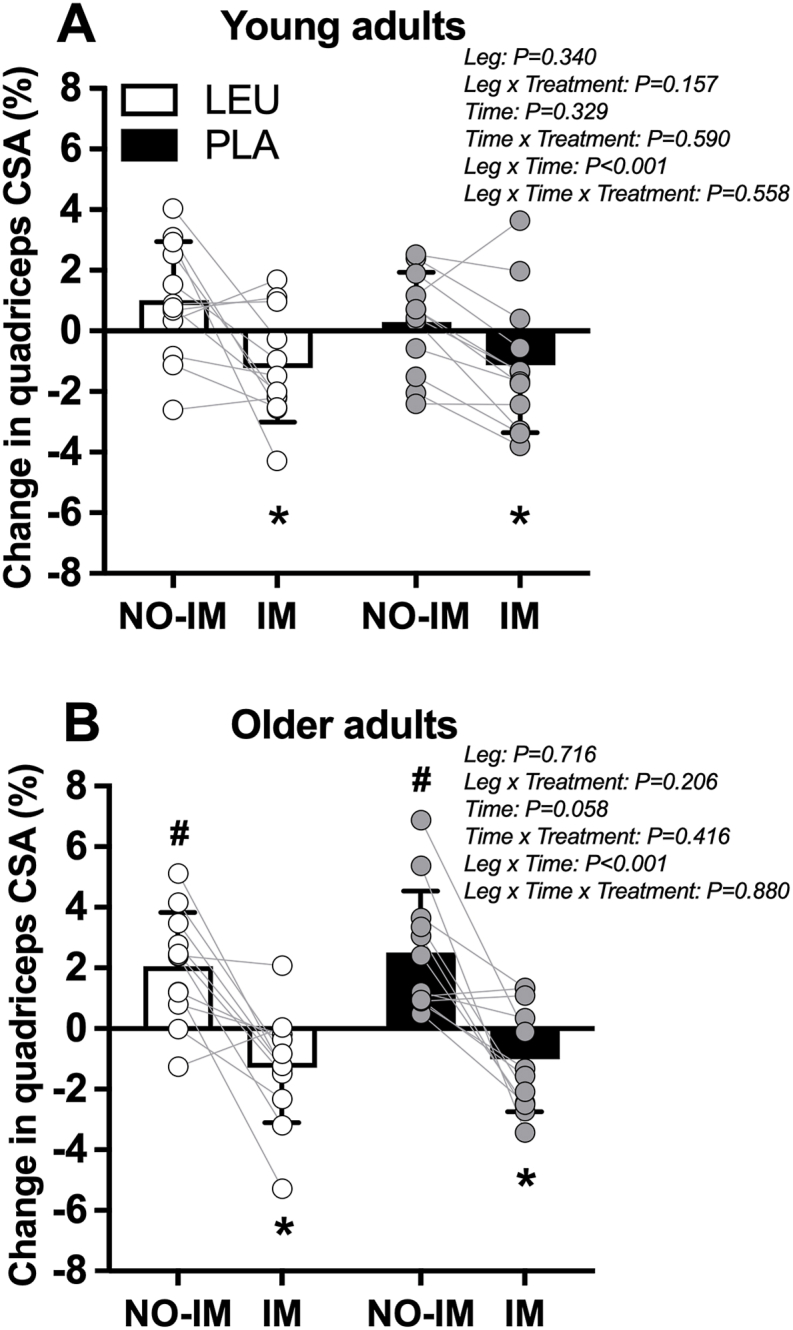
Change in quadriceps muscle CSA (%) in young (A) and older adult (B) study participants who supplemented with 5 g of leucine (LEU) or 5 g of carbohydrate (PLA) 3 × daily with each main meal during 3 d of unilateral knee immobilization by means of a full leg cast. Values represent means ± SD and individual participant data, *n* = 12 per treatment group in each age group. Data were analyzed separately in young and older adult study participants with a 3-factor (leg × time × treatment) repeated-measures ANOVA. Bonferroni post hoc tests were performed to determine the difference between time-points within each leg and the difference between legs within each time-point. ∗Significantly different from Pre (0%) within the IM leg. #Significantly different from Pre (0%) within the NO-IM leg. ANOVA, analysis of variance; CSA, cross-sectional area; IM, immobilized leg; LEU, group of adult study participants who supplemented with 5 g of leucine 3 × daily with each main meal during 3 d of unilateral knee immobilization by means of a full leg cast; NO-IM, nonimmobilized leg; PLA, group of adult study participants who supplemented with 5 g of carbohydrate 3 × daily with each main meal during 3 d of unilateral knee immobilization by means of a full leg cast.

Quadriceps muscle CSA (mm^2^) compared between LEU and PLA treatments in young and older adult participants is shown as percent (%) change in Figure 5A–B. In young adults, there was a leg × time interaction (*P* < 0.001), whereby quadriceps muscle CSA (mm^2^) was lower postimmobilization (total: 7076 ± 1129 mm^2^) compared with preimmobilization (total: 7162 ± 1148 mm^2^) in the IM leg [difference: –86 (95% CI: –150, –22) mm^2^; *P* = 0.011]. There was no difference postimmobilization compared with preimmobilization in the NO-IM leg (*P* = 0.138). There was no effect of treatment (treatment: *P* = 0.374), leg × treatment interaction (interaction: *P* = 0.157), or leg × treatment × time interaction (interaction: *P* = 0.558).

In older adults, there was a leg × time interaction (*P* < 0.001), whereby quadriceps muscle CSA (mm^2^) was lower postimmobilization (total: 5750 ± 1096 mm^2^) compared with preimmobilization (total: 5813 ± 1092 mm^2^) in the IM leg [difference: –64 (95% CI: –108, –19) mm^2^; *P* = 0.007]. Quadriceps muscle CSA (mm^2^) was greater postimmobilization (total: 5881 ± 1118 mm^2^) compared with preimmobilization (total: 5756 ± 1119 mm^2^) in the NO-IM leg [difference: 125 (95% CI: 80, 170) mm^2^; *P* < 0.001]. There was no effect of treatment (treatment: *P* = 0.998), leg × treatment interaction (interaction: *P* = 0.206), or leg × treatment × time interaction (interaction: *P* = 0.880).

Quadriceps muscle CSA (mm^2^) compared between young and older adult participants demonstrated a main effect of age group (*P* < 0.001). Specifically, quadriceps muscle CSA (mm^2^) was lower in older (total: 5800 ± 1143 mm^2^) compared with young (total: 7157 ± 1143 mm^2^) adults [difference: –1357 (95% CI: –2021, –693) mm^2^; *P* < 0.001]. There was also a leg × time interaction (*P* < 0.001) whereby quadriceps muscle CSA (mm^2^) was lower postimmobilization (total: 6413 ± 1288 mm^2^) compared with preimmobilization (total: 6488 ± 1301 mm^2^) in the IM leg [difference: –75 (95% CI: –112, –38) mm^2^; *P* < 0.001]. Quadriceps muscle CSA (mm^2^) was greater postimmobilization (total: 6548 ± 1371 mm^2^) compared with preimmobilization (total: 6466 ± 1406 mm^2^) in the NO-IM leg [difference: 83 (95% CI: 49, 116) mm^2^; *P* < 0.001]. There was no leg × age group interaction (interaction: *P* = 0.765), or leg × time × age group interaction (interaction: *P* = 0.153). Neither the absolute (young: –86 ± 148; older: –64 ± 104 mm^2^; *P* = 0.545) or relative (young: –1.2 ± 2.0; older: –1.1 ± 1.7%; *P* = 0.952) decline in quadriceps muscle CSA in the IM leg was different between younger and older adults.

### Correlation analysis

**FIGURE 6:**
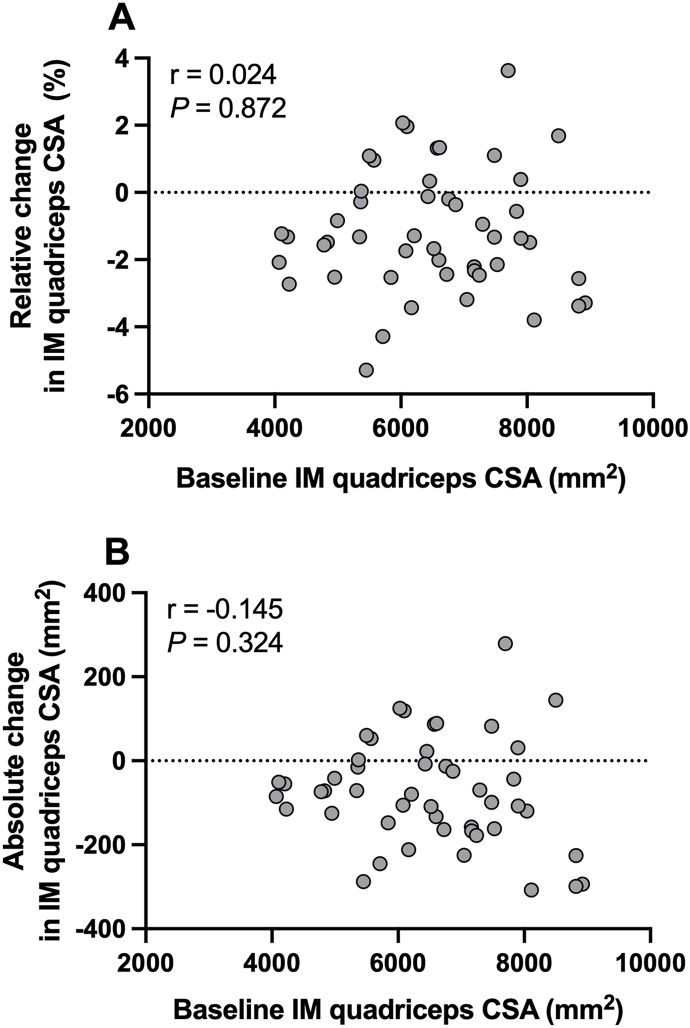
Pearson correlation coefficient analysis of the association between the relative change in the IM quadriceps muscle CSA (%) (A) and the absolute change in the IM quadriceps muscle CSA (mm^2^) (B) in study participants during 3 d of unilateral knee immobilization by means of a full leg cast, and baseline quadriceps muscle CSA (mm^2^) in the IM before immobilization. Values represent individual participant data, *n* = 48 total. Data were collapsed across age groups (young and older adults) and treatment groups who supplemented with 5 g of leucine or 5 g of carbohydrate 3 × daily with each main meal during 3 d of unilateral knee immobilization by means of a full leg cast. CSA, cross-sectional area; IM, immobilized leg.

Pearson correlation coefficient analysis of the relationship between baseline quadriceps muscle CSA (mm^2^) and both the relative (%) and absolute (mm^2^) changes in quadriceps muscle CSA in the IM leg, combined across both young and older participants, is shown in Figure 6A–B. Pearson correlation coefficient analysis demonstrated no association between baseline quadriceps muscle size CSA (mm^2^), and either the relative (%), or absolute (mm^2^), changes in quadriceps muscle CSA in the IM leg after 3 d of immobilization.

## Discussion

In the present study, we evaluated the effect of free leucine supplementation with each main meal (LEU: 5 g, 3 × daily) on daily MPS rates and changes in quadriceps muscle size (CSA) during 3 d of unilateral knee immobilization in both young and older adults as compared with supplementation with an energy-matched amount of carbohydrate (PLA: 5 g, 3 × daily). The principal finding of this study was that LEU supplementation did not attenuate the decline in daily MPS rates or preserve leg muscle mass during short-term limb immobilization in either young or older adults. Specifically, rates of MPS were reduced by ∼15% and ∼23% in the IM compared with the NO-IM leg of young and older adults; however, there were no differences between LEU and PLA treatments in either age group. The decline in daily MPS rates was accompanied by a ∼1.2% and ∼1.1% decrease in quadriceps CSA in the IM leg in both young and older adults, respectively, with no differences between treatments in either age group.

It has been well established that periods of physical inactivity or muscle disuse (e.g., limb immobilization due to casting or bed rest) lower postabsorptive MPS rates [7,8,[39], [40], [41]], and induce anabolic resistance to dietary protein/amino acid provision [7,9,42], thereby resulting in the loss of skeletal muscle mass. The disuse-induced decline in postabsorptive and postprandial MPS rates can occur well within 2 d in response to unilateral knee immobilization in young males [43]. In addition, reductions in leg muscle mass (∼0.85%/d) have been reported to occur in response to <2 d of knee immobilization in young males [5]. In the present study, we applied deuterium oxide (^2^H_2_O) to assess daily MPS rates and demonstrate that 3 d of unilateral leg immobilization resulted in reduced daily MPS rates in the IM in both young (by ∼15%) and older (by ∼23%) adults when compared with the NO-IM leg (Figure 4A–B). The decline in daily MPS rates, which integrates both postabsorptive and postprandial MPS responses over the 3-d immobilization period, was accompanied by a reduction in quadriceps CSA in both the young (∼1.2% or ∼0.40%/d) and older (∼1.1% or ∼0.37%/d) adults. The ∼15% decline in daily MPS rates and ∼1.2% decrease in quadriceps CSA in the young adults are in line with the findings of Kilroe et al. [44], who reported a ∼16% decline in daily MPS rates and a ∼1.7% decline in MRI-derived thigh muscle volume in response to 2 d of unilateral knee immobilization in young males. Similarly, the ∼23% decline in daily MPS rates and ∼1.1% decrease in quadriceps CSA in older adults observed in the present study are comparable to the findings of Smeuninx et al. [45], who observed a ∼21% decline in MPS rates and a 0.7% decline in quadriceps CSA in response to 5 d of inpatient bed rest in older males.

Given the absence of differences between LEU and PLA treatments in the young or older adults, we pooled the data across treatments and compared the impact of 3 d of unilateral knee immobilization on daily MPS rates between the young and older adults (Figure 4A–B). Mixed muscle MPS rates (%/d) were reduced in the IM leg compared with the NO-IM leg in both the young and older adults, but the decline in muscle MPS rates was greater in the older adults. Tanner et al. [10] have directly compared changes in MPS rates in response to a period of disuse between young and older adults. The authors reported that 5 d of bed rest resulted in a blunted stimulation of postprandial mixed MPS rates in response to EAA ingestion in older, but not young adults. Results from the present study seem to align with those of Tanner et al. [10], and suggest that older adults experience a greater suppression of MPS rates in response to short-term muscle disuse when compared with young adults.

It has been proposed [25,[46], [47], [48]] that successive episodes of skeletal muscle disuse during the life course may compound age-related muscle loss. Furthermore, it has been suggested that older adults are more susceptible to skeletal muscle disuse atrophy when compared with young adults [49]. However, whether there are age-related differences in the susceptibility of skeletal muscle to disuse atrophy is unclear as studies have reported both reduced [[26], [27], [28], [29]], and greater [10,30,31] muscle loss in older compared with young adults over a period of muscle disuse. A recent systematic review [50] incorporating disuse periods spanning 4 to 14 d concluded that there are no differences in the rate of muscle atrophy in response to immobilization in older compared with young adults, and, therefore, older adults are not more susceptible to disuse atrophy. Results from the present short-term leg immobilization study seem to align with this conclusion [50], showing no age-related differences in muscle atrophy (either absolute or relative) in response to 3 d of knee immobilization in older compared with young adults.

A potential complicating factor in evaluating age-related differences in the susceptibility of skeletal muscle to disuse atrophy, is age-related differences in initial (i.e., baseline preimmobilization) muscle mass, as the rate and/or magnitude of muscle loss consequent to immobilization may relate to initial muscle mass. For example, Suetta et al. [26], reported a correlation (*r* = –0.669) in young males (but not older males) between baseline quadriceps muscle volume and the decrease in muscle volume after immobilization, whereby greater muscle volume at baseline was associated with a greater relative decline in muscle volume after immobilization. Although other studies [5,27,29] have also reported that larger initial muscle size is associated with a greater magnitude of disuse-induced atrophy, other studies with a larger sample size [51], including the present study, have not. Overall, our data clearly illustrate the deleterious impact of even very short-term periods of muscle disuse on daily MPS rates and skeletal muscle size in both young and older adults.

Postprandial increases in MPS rates appear to be dependent on an increase in essential, rather than nonessential, amino acid availability [[52], [53], [54]]. Of the essential amino acids, the branched-chain amino acid leucine is unique in its capacity to activate the mTORC1 intracellular signaling cascade, a pathway known to upregulate mRNA translation initiation and, as such, stimulate MPS rates [14]. In humans, leucine administration alone (i.e., in the absence of additional exogenous amino acid substrate) can robustly stimulate MPS rates in young adults [15,55]. Furthermore, the addition of leucine to a dose of protein or amino acids has been shown to further augment the postprandial rise in MPS rates [[16], [17], [18], [19],^56^]. Therefore, consumption of free leucine with each main meal (i.e., leucine-enriched meal ingestion) may represent an effective nutritional strategy to compensate for a decline in basal and/or postprandial MPS rates during a period of disuse, and as such, preserve skeletal muscle mass. In support of this notion, English et al. [57] reported that leucine supplementation (0.06 g/kg/meal) reduced DXA-derived whole-body lean mass loss after 7-, but not 14-d of bed rest in middle-aged adults when compared with supplementation with an isonitrogenous alanine supplement. In a follow-up study, the same group [58] again observed a reduction in DXA-derived leg lean mass loss after leucine supplementation (0.06 g/kg/meal) throughout 7 d of bed rest in older adults. These results imply that free leucine supplementation may represent an effective nutrition-based therapeutic strategy to partially preserve skeletal muscle mass during relatively short-term (i.e., ≤7 d) muscle disuse in both middle-aged and older adults. In the present study, we provided 5 g leucine (LEU) or an equivalent amount of carbohydrate (PLA) 3 × daily with each main meal during 3 d of unilateral leg (knee) immobilization to a group of young and older adults to assess whether free leucine supplementation could overcome immobilization-induced anabolic resistance, increase daily MPS rates, and as such, preserve leg muscle mass during short-term immobilization. After immobilization, we observed a substantial decline in daily MPS (Figure 3A–D) and a concomitant decline in leg muscle size (Figure 4A–B) in both age groups. However, there were no differences in MPS rates or decline in muscle CSA between the LEU and PLA supplemented groups in both the young and older adults. Therefore, our data demonstrate no protective effect of free leucine supplementation on daily MPS rates or skeletal muscle size during short-term unilateral leg (knee) immobilization in either young or older adults. These findings are consistent with previous work from our laboratory [59], showing that leucine supplementation with each main meal (2.5 g leucine, 3 × daily) does not attenuate the decline in quadriceps muscle CSA in response to 7-d of unilateral knee immobilization in young males. Our findings also align with those of Edwards et al. [60], who reported that free leucine supplementation (5 g leucine, 3 × daily) with each main meal did not prevent disuse-induced “anabolic resistance” to ingested dietary protein (20 g milk protein) or attenuate the decline in leg fat-free mass in response to 7 d of unilateral knee immobilization in young males. The discrepancy between studies with regards to the proposed protective effect of leucine supplementation on muscle loss during a period of disuse is unclear but may be attributed to the applied disuse model (i.e., systemic bed rest compared with localized limb immobilization), age of the participants (young, middle-aged, and older adults), and/or dose of free leucine (0.06 g/kg/meal compared with absolute doses 2.5–5.0 g per meal).

In addition to studies evaluating the effects of free leucine supplementation, other studies have evaluated the capacity of a more complete mixture of essential amino acids [61], various sources of supplemental isolated proteins [[62], [63], [64]], a higher-protein (1.6 g protein/kg body mass/d) diet [4], and a specific mixture of essential and nonessential amino acids (i.e., AXA2678) [65], to maintain MPS rates [4,61,63,64], and/or preserve skeletal muscle mass [4,[61], [62], [63], [64], [65]], during periods of muscle disuse. All of the nutritional interventions from these studies were associated with increased leucine provision, but their results were somewhat discrepant. For example, Paddon-Jones et al. [61] reported that ingestion of 16.5 g of essential amino acids with 30 g sucrose 3 × daily during 28-d of bed rest maintained MPS rates and leg lean mass in young males. Alternatively, our laboratory has previously shown that dietary protein supplementation (∼20 g, 2 × daily) did not preserve skeletal muscle mass in response to 5 d of unilateral leg (knee) immobilization in older males [62]. In support of this finding, a recent systematic review and meta-analysis [66] evaluating the effect of protein or amino acid provision on immobilization-induced muscle atrophy in healthy adults revealed no effect (standardized mean difference: 0.2; 95% CI: –0.18, 0.57, *P* = 0.31) of protein/amino acid interventions in preventing disuse-induced muscle atrophy. Future studies exploring preventative countermeasures against disuse-induced declines in MPS rates and muscle size should encompass both leucine/protein supplementation alongside contractile stimuli (e.g., exercise, neuromuscular electrical stimulation) as a strategy to protect skeletal muscle size and metabolic function.

In conclusion, leucine supplementation does not attenuate the decline in daily skeletal MPS rates or preserve leg muscle mass during short-term limb immobilization in either young or older adults. Both young and older adults are susceptible to a rapid decline in daily MPS rates during a short period of limb immobilization, resulting in a significant decline in leg muscle mass.

## Author contributions

The authors’ responsibilities were as follows – TAC-V, LJCvL: designed the research; TAC-V, PJMP, JSJS, GNM-N, JMS: conducted the research; LJCvL: provided essential materials; TAC-V, PJMP, SHMG, CJF, JMS, JPBG, APG, WKWHW: analyzed data; TAC-V, PJMP: performed the statistical analysis; TAC-V, PJMP, LJCvL: wrote the manuscript; TAC-V, PJMP, LJCvL: had primary responsibility for final content; and all authors: read and approved the final manuscript.

## Data availability

Data described in the manuscript will be made available upon request pending application to and approval from the corresponding author.

## Declaration of generative AI and AI-assisted technologies in the writing process

None used.

## Funding

TAC-V was supported by a European Commission Horizon 2020 Marie Skłodowska-Curie Individual Fellowship (659739-REALISM) and a Natural Sciences and Engineering Research Council of Canada (NSERC) Postdoctoral Fellowship (PDF-471384-2015).

## Conflict of interest

TAC-V reports financial support was provided by Natural Sciences and Engineering Research Council of Canada. TAC-V reports financial support was provided by European Commission. LJCvL reports a relationship with Friesland Campina that includes: consulting or advisory, funding grants, and speaking and lecture fees. LJCvL reports a relationship with Arla Foods Ingredients that includes: consulting or advisory, funding grants, and speaking and lecture fees. If there are other authors, they declare that they have no known competing financial interests or personal relationships that could have appeared to influence the work reported in this paper.
