# Supplementary material for: Leucine supplementation does not attenuate the decline in daily muscle protein synthesis rates or preserve leg muscle mass during leg immobilization in young or older adults: a double-blind randomized trial
Source: Am J Clin Nutr. 2026 Jan 22;123(4):101205. doi: 10.1016/j.ajcnut.2026.101205 (PMC13084588; doi:10.1016/j.ajcnut.2026.101205)
Supplement: Multimedia component 1 [file mmc1.docx]

| **Supplemental Table 1.** Nutritional intake and step-count of young and older adults over 3 days before onset of the intervention (Baseline), and during 3 days of unilateral knee immobilization supplemented with either 5 g of carbohydrate (PLA) or 5 g of leucine (LEU) 3 × daily with each main meal. | | | | | | | | | | | | | |
| --- | --- | --- | --- | --- | --- | --- | --- | --- | --- | --- | --- | --- | --- |
| **Young** | | | | | | | | | | | | | |
|  | Baseline | | | | | | Immobilization | | | | | |  |
|  | PLA | | | LEU | | | PLA | | | LEU | | | *P*-value |
| Energy intake (MJ) | 9.23 | ± | 2.69 | 8.32 | ± | 1.27 | 8.95 | ± | 2.01 | 8.41 | ± | 1.11 | Time: 0.790  Treatment: 0.331  Interaction: 0.588 |
| Carbohydrates (g) | 279 | ± | 92 | 244 | ± | 57 | 269 | ± | 101 | 227 | ± | 47 | Time: 0.387  Treatment: 0.209  Interaction: 0.804 |
| Fat (g) | 85 | ± | 32 | 77 | ± | 11 | 78 | ± | 25 | 85 | ± | 18 | Time: 0.889  Treatment: 0.930  Interaction: 0.185 |
| Protein (g) | 82 | ± | 19 | 81 | ± | 20 | 89 | ± | 19 | 85 | ± | 26 | Time: 0.174  Treatment: 0.723  Interaction: 0.744 |
| Protein (g/kg BM) | 1.2 | ± | 0.4 | 1.2 | ± | 0.3 | 1.3 | ± | 0.5 | 1.3 | ± | 0.4 | Time: 0.177  Treatment: 0.824  Interaction: 0.890 |
| Carbohydrates (En%) | 50 | ± | 5 | 48 | ± | 6 | 50 | ± | 11 | 45 | ± | 7 | Time: 0.432  Treatment: 0.247  Interaction: 0.457 |
| Fat (En%) | 34 | ± | 7 | 35 | ± | 6 | 33 | ± | 8 | 38 | ± | 7 | Time: 0.705  Treatment: 0.238  Interaction: 0.253 |
| Protein (En%) | 16 | ± | 5 | 16 | ± | 4 | 17 | ± | 4 | 17 | ± | 4 | Time: 0.194  Treatment: 0.947  Interaction: 0.594 |
| Step-count | 7910 | ± | 3258 | 4676 | ± | 3052 | 1858 | ± | 856 | 1564 | ± | 647 | **Time: <0.001**  **Treatment: 0.029**  **Interaction: 0.030** |

| **Older** | | | | | | | | | | | | | |
| --- | --- | --- | --- | --- | --- | --- | --- | --- | --- | --- | --- | --- | --- |
|  | Baseline | | | | | | Immobilization | | | | | |  |
|  | PLA | | | LEU | | | PLA | | | LEU | | | *P*-value |
| Energy intake (MJ) | 7.75 | ± | 1.89 | 8.61 | ± | 2.29 | 7.77 | ± | 1.99 | 7.96 | ± | 2.83 | Time: 0.219  Treatment: 0.572  Interaction: 0.184 |
| Carbohydrates (g) | 209 | ± | 53 | 244 | ± | 78 | 202 | ± | 61 | 233 | ± | 103 | Time: 0.406  Treatment: 0.274  Interaction: 0.829 |
| Fat (g) | 76 | ± | 25 | 84 | ± | 23 | 79 | ± | 26 | 74 | ± | 28 | Time: 0.347  Treatment: 0.889  Interaction: 0.123 |
| Protein (g) | 85 | ± | 18 | 81 | ± | 21 | 85 | ± | 17 | 77 | ± | 27 | Time: 0.546  Treatment: 0.442  Interaction: 0.613 |
| Protein (g/kg BM) | 1.2 | ± | 0.2 | 1.1 | ± | 0.3 | 1.2 | ± | 0.2 | 1.1 | ± | 0.3 | Time: 0.571  Treatment: 0.519  Interaction: 0.463 |
| Carbohydrates (En%) | 45 | ± | 6 | 47 | ± | 6 | 44 | ± | 8 | 48 | ± | 9 | Time: 0.743  Treatment: 0.308  Interaction: 0.368 |
| Fat (En%) | 36 | ± | 6 | 37 | ± | 5 | 37 | ± | 6 | 36 | ± | 8 | Time: 0.889  Treatment: 0.951  Interaction: 0.243 |
| Protein (En%) | 19 | ± | 3 | 16 | ± | 2 | 19 | ± | 3 | 17 | ± | 3 | Time: 0.686  **Treatment: 0.016**  Interaction: 0.694 |
| Step-count | 7917 | ± | 3590 | 8263 | ± | 2739 | 1073 | ± | 482 | 1335 | ± | 491 | **Time: <0.001**  Treatment: 0.670  Interaction: 0.946 |
| ^1^Values are mean ± SD.  ^2^Young: data available from *n*=22.  ^3^Older: data available from *n*=23.  ^4^Abbreviations: kJ, kilojoule; BM, body mass. | | | | | | | | | | | | | |
