# Supplementary material for: Leucine supplementation does not attenuate the decline in daily muscle protein synthesis rates or preserve leg muscle mass during leg immobilization in young or older adults: a double-blind randomized trial
Source: Am J Clin Nutr. 2026 Jan 22;123(4):101205. doi: 10.1016/j.ajcnut.2026.101205 (PMC13084588; doi:10.1016/j.ajcnut.2026.101205)
Supplement: Supplementary file 2 [file mmc2.docx]

Leucine supplementation does not attenuate the decline in daily muscle protein synthesis rates or preserve leg muscle mass during leg immobilization in young or older adults: a double-blind randomized trial

Tyler A. Churchward-Venne
